# Effects of underweight, overweight, and obesity on the body growth of preschoolers

**DOI:** 10.3389/fpubh.2024.1454436

**Published:** 2024-12-18

**Authors:** Jiebo Chen, Dongmei Luo, Xue Fan

**Affiliations:** ^1^Department of Sports Science, Nantong University, Nantong, China; ^2^Department of Kinesiology, Beijing Sport University, Beijing, China; ^3^Department of Physical Education, Anhui University of Technology, Ma’anshan, China

**Keywords:** underweight, overweight, obesity, body mass index, body shape

## Abstract

**Objective:**

This study aimed to investigate the impact of underweight, overweight, and obesity on the growth and development of preschoolers by comparing body shape characteristics across different weight statuses.

**Methods:**

A total of 729 preschoolers (5.2 ± 0.83 years, 53.8% boys) from three kindergartens were assessed for 11 different body shape measurements. Two-way ANOVA was employed to examine BMI variations across different ages and sexes. Discriminant analysis was utilized to identify body shape measurements correlated with BMI, and one-way ANOVA was conducted to compare the body shape differences among preschoolers with varying BMI.

**Results:**

(1) There was no significant interaction effect of gender and age on BMI (*F* = 1.602, *p* = 0.173). Additionally, neither the main effect of age (*F* = 1.461, *p* = 0.228) nor the main effect of sex (*F* = 0.905, *p* = 0.345) was significant. (2) The results of the stepwise discriminant analysis showed that chest circumference, calf length, calf circumference, foot length, and width between greater trochanters entered the discriminant model, with the three discriminant functions explaining 95.8, 3.1, and 1.1% of variance, respectively. (3) Compared to their normal-weight counterparts, obese preschoolers displayed significantly larger measurements in chest circumference, width between greater trochanters, calf circumference, calf length, and foot length (*p* < 0.05). Overweight preschoolers also exhibited larger chest and calf circumferences, and width between greater trochanters (*p* < 0.05), while underweight children showed lagging development in various body shape measurements (*p* < 0.05).

**Conclusion:**

Variations in BMI were significantly correlated with preschoolers’ body shape which included chest circumference, calf length, calf circumference, foot length, and the distance between the greater trochanters. Overweight and obese preschoolers experienced faster body growth; conversely, underweight preschoolers often showed delayed growth. This underscores that the underweight group also merits attention and concern.

## 1 Introduction

With the rapid development of technology and economy, sedentary lifestyles have become increasingly prevalent. The general lack of physical activity exacerbates the risk of obesity (1). Now considered one of the most pressing public health challenges of the 21st century, obesity is no longer confined to developed nations. Current statistics indicate that childhood obesity rates are also increasing in developing countries (2, 3). In China, the rate of overweight and obesity among children under the age of 6 has already reached 10.4% (4). This number should not be overlooked, as it portends a significant burden on health and socioeconomics in the future.

The consequences of obesity are multifaceted. Studies have shown that obese preschoolers experience poorer physical fitness, diminished gross motor skills, an increased risk of fractures and early markers of cardiovascular disease (5–7). Additionally, childhood obesity has adverse psychosocial consequences and lowers educational attainment (8, 9). Moreover, children with excess weight are more likely to become obese adults (10). Despite the ample research on these aspects, there is a notable lack of focus on how overweight and obesity affect body growth.

During children’s growth, their body shape experiences notable changes. By analyzing these changes, researchers assess the impact of environmental and genetic factors on human biological adaptation (11, 12). This assessment heavily relies on anthropometric measurements, like limb circumferences and lengths, which helps understand growth and body composition (13). For instance, measuring the thighs and calves’ circumference aids in evaluating muscle growth (14), providing proxy indicators for muscle mass (15). Moreover, body shape significantly influences people’s psychological wellbeing, especially among those adolescents or children with obesity (16). Research indicates a strong correlation between body shape concerns and general psychological distress (17, 18). Overweight and obese children, in comparison to their peers with a healthy weight, often display heightened concern over their body shape (19).

Moreover, the issue of underweight remains a significant concern, although its prevalence ranges between 3 and 8%, which is lower than that of overweight (20, 21). Childhood underweight is often a long-term condition that typically signals future health risks, including various diseases (22). Research indicates a direct correlation between low Body Mass Index (BMI) in children and heightened risks of coronary heart disease (23) and depression (24). Therefore, this study not only examines the body shape of overweight and obese children but also focuses on the underweight children.

While numerous studies have explored the multifaceted impacts of underweight, overweight, and obesity, research on the specific effects of different weight statuses on body growth (such as lower limb length, foot length, and pelvic width) is relatively lacking, especially among preschoolers. Therefore, the aim of this study is to investigate the differences in body shape among Chinese children aged 4 to 6 with different weight statuses. This not only provides new scientific evidence on the relationship between weight and growth in preschoolers but also offers a basis for formulating public health policies and designing early intervention measures, thereby helping to better identify and prevent the health risks associated with abnormal weight in preschoolers.

## 2 Materials and methods

### 2.1 Participants

A total of 765 children were initially recruited by convenience from three kindergartens in Beijing, China. The recruitment and testing of these children occurred in October and November of 2021, 2022, and 2023, with 152, 317, and 296 children recruited in those 3 years, respectively. Due to illnesses, 36 preschoolers were unable to participate in the experiment, resulting in a final sample size of 729 (boys: 53.8%, 5.2 ± 0.86 years; girls: 46.2%, 5.3 ± 0.77 years). All participants were healthy preschoolers without any cognitive or physical developmental disorders. Parents of all participants signed the Parental Informed Consent Form to indicate their consent to participate in the study.

We employed G.Power 3.1.9.7 for *post hoc* analysis to evaluate the statistical power of the sample size. The statistical analysis selected ANOVA (Fixed effects, special, main effects, and interactions) from the F test. The input parameters were as follows: Effect size *f* = 0.25 (medium effect size) (25), *α* = 0.05, total sample size = 729, numerator degrees of freedom = 2, and the number of groups = 6 (sex*2 and age*3). The output parameters showed a Power (1-*β*) of 0.999, which suggests that the sample size in this study possesses enough statistical power, satisfying the research criteria.

### 2.2 Instruments and procedure

Height and weight were measured according to the early childhood section of the China National Physical Fitness Determination Standard Manual, formulated by the State General Administration of Sports (26). Body Mass Index (BMI), widely accepted as a measure of overweight and obesity in children older than 2 years, was calculated with the formula: BMI = weight/height^2^. The BMI cut-off points for the classification of underweight, overweight and obesity are established by the International Obesity Task Force (IOTF) (27, 28), The BMI cut-off points (kg/m^2^) for 4-year-old children are 14.43, 17.55, and 19.29 for boys and 14.19, 17.28, and 19.15 for girls. For 5-year-old children are 14.21, 17.42, and 19.30 for boys and 13.94, 17.15, and 19.17 for girls. For 6-year-old children are 14.07, 17.55, and 19.78 for boys and 13.82, 17.34, and 19.65 for girls.

**Figure 1:**
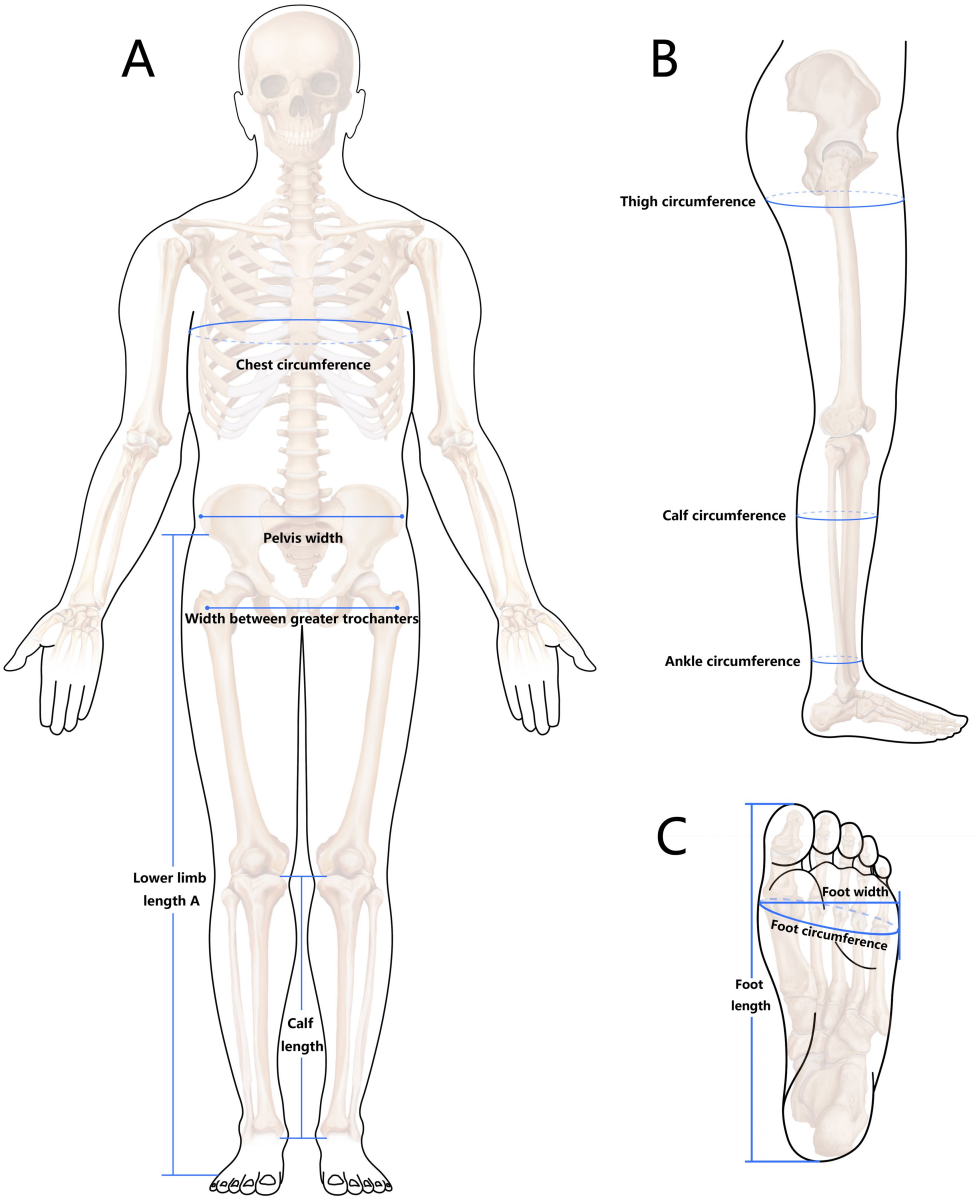
A schematic diagram of body shape measurements.

Body shape was assessed using the measurements from the Chinese Sports Measurement and Evaluation Standard (29). These measurements included length, width and circumference parameters. The length measurements included lower limb length, calf length and foot length; the width measurements included pelvic width, width between greater trochanters and foot width; and the circumference measurements included chest, thigh, calf, ankle and foot circumferences. Definitions and instruments used for these measurements are detailed in Table 1 and Figure 1. The anthropometric instruments used were sourced from Leatech in China.

**Table 1:** Definition and instrument for body shape measurements.

Measurement		Definition	Instrument (model)
Length	Lower limb length A	The vertical distance from the point of the left anterior superior iliac spine to the floor	Long martin anthropometer (D403008)
	Calf length	The vertical distance from the superior edge of the medial tibial condyle to the tip of the medial ankle	Short martin anthropometer (D403008)
	Foot length	The vertical distance between the point of the heel and the farthest point of the tip of the toe	Segmometer (CC01)
Circumference	Chest circumference	The circumference from the lower margin of the subscapular Angle to the nipple point	Tape measure (NC70170)
	Thigh circumference	The circumference of the thigh at the transverse hip line	Tape measure (NC70170)
	Calf circumference	The circumference of the thickest part of the calf	Tape measure (NC70170)
	Ankle circumference	The circumference of the thinnest part of the ankle	Tape measure (NC70170)
	Foot circumference	The circumference of the foot from the first metatarsal to the fifth metatarsal bone	Tape measure (NC70170)
Width	Pelvis width	The distance between the tubercle of iliac crest (widest part of the pelvis)	Sliding caliper (121230)
	Width between greater trochanters	The distance between the two sides of the large greater trochanter	Sliding caliper (121230)
	Foot width	The width of the foot from the first metatarsal to the fifth metatarsal bone	Vernier caliper (98604)

All tests were conducted in the gymnastics classroom of the respective schools and lasted approximately 20–25 min per child. The tests were administered by four trained research assistants in a specific sequence: the first assistant measured height and weight, the second focused on length, the third on width, and the fourth on circumference measurements related to body shape. Each parameter was measured twice by the same assistant, and the results were averaged for greater accuracy.

### 2.3 Statistical analyses

SPSS 17.0 was employed for data analysis, with a preset significance level of 0.05. In this study, two-way ANOVA was employed to assess the effects of age and sex on preschooler’s BMI, with the Kolmogorov–Smirnov test for normality and Levene’s test for variance homogeneity. Stepwise discriminant analysis was utilized to identify body shape measurements that were highly correlated with BMI. In this analysis, children were grouped into four categories: underweight, normal weight, overweight, and obese. These body shape measurements served as independent variables, and the Wilks’ Lambda method was applied for stepwise discrimination of variables. Criteria for variable entry and exclusion were set at *F* > 3.84 and *F* < 2.71, respectively. Lastly, one-way ANOVA was conducted to compare body shape characteristics among preschoolers of different BMI levels, *post hoc* comparisons were conducted using the LSD method when homogeneity of variance was satisfied and Tamhane’s T2 method when heterogeneity of variance was observed.

## 3 Results

### 3.1 Characteristics of BMI changes in preschoolers

The characteristics of the participants are shown in Table 2. Statistical analysis indicated that the data in this study were approximately normally distributed (*p* > 0.05) and exhibited homogeneity of variance (*p* = 0.079). The analysis revealed no significant interaction effect of gender and age on BMI (*F* = 1.602, *p* = 0.173). Additionally, neither the main effect of age (*F* = 1.461, *p* = 0.228) nor the main effect of sex (*F* = 0.905, *p* = 0.345) was significant. Thus, no distinction is made between age and sex in the subsequent analysis.

**Table 2:** The characteristics of the participants (Mean ± SD).

Age	*N*		Height (cm)		Weight (kg)		BMI(kg/m^2^)	
	Boys	Girls	Boys	Girls	Boys	Girls	Boys	Girls
4	129	113	103.35 ± 6.18	102.18 ± 6.23	16.57 ± 2.70	15.83 ± 4.54	15.47 ± 1.68	14.86 ± 3.07
5	142	124	110.95 ± 4.84	108.67 ± 6.88	19.78 ± 2.64	18.57 ± 3.89	16.02 ± 1.26	15.55 ± 1.58
6	121	100	118.03 ± 7.48	114.50 ± 6.06	22.48 ± 5.05	20.74 ± 3.17	16.11 ± 3.16	15.80 ± 2.78

### 3.2 Screening for body shape measurements associated with BMI

Stepwise regression was used to establish discriminant functions to screen the measurements related to BMI, and the results indicated that chest circumference, calf length, calf circumference, foot length, and width between greater trochanters entered the model (see Table 3).

**Table 3:** Variables entered into the model using stepwise regression.

	Measurements	Wilks’ Lambda			
		F	df1	df2	*P*
1	Chest circumference	30.609	3	165.045	<0.001
2	Calf length	19.085	6	245.688	<0.001
3	Calf circumference	8.187	9	314.947	0.002
4	Foot length	9.514	12	450.003	<0.001
5	Width between greater trochanters	7.778	15	473.163	0.007

Three discriminant functions were established through stepwise regression. The first function accounted for 95.8% of the variance, the second for 98.9%, and the third function explained 100% of the variance. All three functions were statistically significant, as shown in Table 4. Based on these findings, chest circumference, calf length, calf circumference, foot length, and width between greater trochanters were identified as key body shape measurements that reflected changes in BMI.

**Table 4:** The eigenvalues of the discriminant function.

Discriminant function	Characteristic root	Equation of interpretation (%)	Total variance (%)	Typical correlation coefficients	*P*
1	1.741	95.8	95.8	0.724	<0.001
2	0.076	3.1	98.9	0.265	<0.001
3	0.030	1.1	100.0	0.057	<0.001

### 3.3 Body shape differences in preschoolers with different weight status

The results of the one-way ANOVA, as detailed in Table 5, revealed significant differences in body shape across weight statuses. Specifically, the obese group had significantly higher measurements in chest circumference, width between greater trochanters, calf circumference, and calf length compared to the underweight, normal and overweight groups. Furthermore, chest circumference, calf circumference, and width between greater trochanters were also significantly higher in the overweight group than in the normal group. Comparatively, the normal group displayed higher measurements in chest circumference, calf circumference, and width between greater trochanters than the underweight group. Additionally, foot length was significantly elevated in the obese group when compared to either the normal or the underweight groups.

**Table 5:** Body shape characteristics of preschoolers with underweight, normal weight, overweight, and obesity (Mean ± SD).

Measurements	Underweight (*N* = 42)	Normal weight (*N* = 564)	Overweight (*N* = 82)	Obesity (*N* = 41)
Chest circumference (cm)	50.86 ± 3.30	53.38 ± 2.98^a^	56.65 ± 2.44^ab^	62.00 ± 3.41^abc^
Calf length (cm)	22.42 ± 1.34	22.94 ± 2.04	23.21 ± 1.32^a^	24.14 ± 2.04^abc^
Calf circumference (cm)	21.69 ± 1.81	22.98 ± 1.39^a^	24.36 ± 1.57^ab^	26.20 ± 2.02^abc^
Foot length (cm)	17.26 ± 0.94	17.35 ± 1.32	17.63 ± 0.90	18.32 ± 1.08^ab^
Width between greater trochanters (cm)	18.64 ± 1.33	19.70 ± 1.09^a^	20.61 ± 1.31^ab^	21.66 ± 1.10^abc^

^a^Marks a significant difference compared with the underweight group (*P* < 0.05); ^b^marks a significant difference compared with the normal group (*P* < 0.05); ^c^marks a significant difference compared with the overweight group (*p* < 0.05).

## 4 Discussion

The present study demonstrated significant variations in specific anthropometric parameters--chest circumference, calf length, calf circumference, foot length, and width between greater trochanters among preschoolers categorized by different BMI levels. These findings underscored the strong association between weight status and the overall body growth in preschoolers.

Our study demonstrated a positive correlation between BMI and calf length, foot length, and width between greater trochanter in preschoolers. These measurements were indicative of growth progress; calf length and foot length served as markers for lower limb and foot bone development, while width between greater trochanter reflected pelvic development. These observations seemed to imply that a higher BMI was linked to accelerated growth and development in preschoolers. Concordant with this finding was a report that obese children exhibited elevated levels of insulin-like growth factor-1 (IGF-1), a hormone known to stimulate bone development (30). However, it is essential to note that although obese children may show advanced bone development, they often possess lower bone mass and density compared to their normal-weight peers, making them more susceptible to fractures (31). Previous research has corroborated this having found that obese children accounted for a higher percentage of cases with distal fractures (32). The length of lower limbs and foot has been shown to be a reliable dimension for estimation of height (33, 34). However, obesity may affect the accuracy of this estimate, as Kain et al. (35) suggested that although obese preschoolers may be taller than normal, there was no significant difference in height between them and normal individuals in late adolescence and adulthood. Therefore, our study suggest that researchers and doctors should rationalize this change in body shape in obese children as it may be temporary and could be linked with other musculoskeletal problems.

Our study indicated that overweight and obese preschoolers have larger chest circumferences. Akaboshi et al. (36) demonstrated that chest circumference during infancy is predictive of obesity in children by the age of three. Therefore, measuring chest circumference might serve as a valuable tool for clinicians in identifying childhood obesity. Additionally, our study observed that overweight and obese preschoolers tend to have higher calf circumferences. Previous research confirmed that the expanded girth observed in overweight and obese preschoolers was primarily attributable to excessive fat accumulation (37). The results in our study suggested that excess fat tended to accumulate more in the chest and calf areas in overweight and obese preschoolers. This accumulation of fat could also potentially impact a preschooler’s motor competence. Studies by Songhua et al. (38) have shown that overweight and obese children exhibited altered gait patterns, including shorter strides and modified knee and ankle angles in the sagittal plane, pointing to compromised lower limb functions. Other investigations have affirmed that overweight and obese preschoolers generally experience delays in the development of fundamental motor skills (39–41). According to Newell’s constraint model of motor development, body shape was an important limiting factor (42). The present study might therefore postulate that the body shape of obese preschoolers could impose certain constraints on their motor development. Exploring the relationship between body shape and motor development in overweight and obese preschoolers could be an important avenue for future research, potentially providing a basis for scientifically designed physical activities for kindergartens.

Not to be overlooked, this study discovered that underweight preschoolers exhibited slower body growth compared to their peers in the normal weight, overweight, and obese groups. Being underweight serves as a substantial marker for malnutrition, a condition that has long-term consequences on children’s survival, cognitive development, and future productivity (43). Consistent with this, other research has reported that underweight preschoolers tended to have poorer physical fitness compared to their normal-weight counterparts (44). Despite economic advancements reducing the prevalence of underweight children under the age of six in China to about 5% (4), improving the growth and development of these young children remains an urgent issue.

This study had some limitations that need to be considered in future research. Firstly, the study included a limited range of body shape measurements. In studies conducted in different populations, there has been a significant association between obesity and metrics like neck circumference (45), waist circumference (46), waist-to-hip ratio (47), and waist-to-height ratio (48). Future studies are encouraged to investigate these measurements in the context of obesity in preschoolers. Secondly, while this study identified measurements such as chest circumference, calf length, calf circumference, foot length, and width between greater trochanters as indicators of overweight, obesity, or underweight in preschoolers, it fell short of defining optimal cut-off values. Future research, especially with a larger cohort, should consider utilizing receiver operating characteristic (ROC) curves to establish these critical values.

## 5 Conclusion

Variations in BMI were significantly correlated with preschoolers’ body shape which included chest circumference, calf length, calf circumference, foot length, and the distance between the greater trochanters. Overweight and obese preschoolers experienced faster body growth; conversely, underweight preschoolers often showed delayed growth. This underscores that the underweight group also merits attention and concern.

## Data Availability

The original contributions presented in the study are included in the article/supplementary material, further inquiries can be directed to the corresponding author.
